# Endogenous and environmental factors that induce DNA replication defects and genomic instability in ER-negative heterozygous BRCA1 cells

**DOI:** 10.1038/s41598-026-46028-5

**Published:** 2026-03-27

**Authors:** Madhura Deshpande, Theodore Paniza, Rebecca Brown, Kate Heslin, Nitya Patel, Advaitha Madireddy, Zev Rosenwaks, Jeannine Gerhardt

**Affiliations:** 1 The Ronald O. Perelman and Claudia Cohen Center for Reproductive Medicine, Weill Cornell Medicine, 515E 71 st Street, New York, 10021 NY USA; 2 Department of Obstetrics and Gynecology, Weill Cornell Medicine, New York, NY USA; 3 Department of Pediatric Hematology/Oncology, Rutgers University, New Brunswick, NJ USA

**Keywords:** Cancer, Cell biology, Genetics, Molecular biology, Oncology

## Abstract

Carriers with a germline mutation in the BRCA1 gene have an increased lifetime risk of breast and ovarian cancer and other malignancies. Cancer initiation is linked to mutagenesis and loss of heterozygosity (LOH) in BRCA1 carriers. Approximately 70% of BRCA1-associated breast cancers are triple negative (TNBC; progesterone (PR), HER2, estrogen receptor (ER)-negative), which were reported to develop from ER/PR-negative luminal progenitor cells. However, the mechanisms and factors inducing carcinogenesis in ER/PR-negative cells carrying a BRCA1 germline mutation are not known. Also, ER/PR-negative breast cancer cells are not responsive to ER hormone therapy, making it challenging to treat TNBC cancers. We investigated ER-negative mammary cells and found that estrogen and estrogen metabolites, which are known to form DNA adducts, inhibit replication fork progression in heterozygous BRCA1^mut/+^ mammary cells. Furthermore, estrogen triggered DNA breaks, large deletions, and cancer-initiating mutations, such as LOH in BRCA1^mut/+^ mammary cells. In addition, we found that one of the most commonly used herbicides in the US, the endocrine-disruptor Atrazine, also hinders replication fork progression and induces genomic instability in these cells. To counteract the genotoxic effect, we tested several dietary compounds and found that Indole-3 carbinol (I3C) prevents the replication stress and reduces genomic instability in BRCA1^mut/+^ mammary cells. In summary, these results reveal that alterations in estrogen metabolism caused by environmental or endogenous factors are genotoxic for ER-negative BRCA1^mut/+^ mammary cells. And our results also show that the dietary compound I3C can prevent estrogen-induced DNA damage, and suggest that I3C can be a potential cancer-preventive therapeutic agent for BRCA1 carriers.

**Supplementary Information:**

The online version contains supplementary material available at 10.1038/s41598-026-46028-5.

## Introduction

Breast cancer is the leading cause of cancer in women in the United States and the third leading cause of cancer death. Germline mutations in one copy of the *BRCA1* or *BRCA2* gene account for the majority of hereditary breast cancers^1^. These mutations are linked to a higher risk of breast cancer (approximately 70–80%) and ovarian cancer (45% for *BRCA1* and 20% for *BRCA2* carriers)^2^. In addition, breast cancer incidence has increased from about 1 in 11 in 1975 to 1 in 8 incidences today. This increase could be explained by several factors, including elevated exposure of BRCA carriers to mutagenic compounds or pollutants. For example, residues from pesticides and growth hormones that have estrogen-like properties or induce estrogen production can be found in food and the water supply^3^. Since it is known that estrogen and estrogen metabolites induce DNA damage in breast epithelial cells^4^, these environmental residues could be very detrimental to BRCA1 carriers.

Cancer development in BRCA1 carriers is connected to DNA mutations and genomic rearrangements in a variety of tumor suppressor genes, such as p53^5,6^. A significant number of BRCA1 breast cancers (90–93%) have been reported to harbor mutations in the second copy of the *BRCA* gene (also known as locus-specific loss of heterozygosity, LOH)^7–10^. However, the mechanisms and factors inducing carcinogenesis, especially in BRCA1 carriers, are not known. Estrogen was shown to induce DNA lesions^4,11,12^ and cause DNA breaks in estrogen receptor (ER)-positive and -negative breast epithelial cells^4^. It was reported previously that estrogen induces R-loop formation and genomic instability in ER-positive cells, but not ER-negative breast epithelial cells^13^. However, 70% of BRCA1-associated breast cancers are triple-negative breast cancer (TNBC; estrogen, progesterone, and HER2 receptor-negative)^14^, and ER-negative mammary cells were indicated to be the origin of TNBC^15^. It is not known if and how estrogen induces genomic instability in ER-negative luminal/progenitor cells, including BRCA1 carrier cells.

Estrogen is suspected to be a carcinogen due to its binding and activation of the ER alpha, and enhancing tumor development in ER-positive cancer cells^16^. However, the role of estrogen in initiating tumorigenesis in human ER-negative mammary cells and in cells carrying a BRCA1 germline mutation is not clearly understood. Since it is known that estrogen metabolites are able to form depurinating DNA adducts, which were detected in vitro and in vivo in model systems^17,18^, women’s breast tissues^19^, and the urine of breast cancer patients^20^, it is suspected that estrogen metabolites could cause point mutations that lead to cancer development. However, so far, estrogen-DNA adducts have not been linked to large deletions that prompt cancer initiation. The formation of depurinating DNA adducts due to an increase in estrogen levels could be especially detrimental for BRCA1 carriers, given their limited BRCA1 protein expression. BRCA1 is involved in DSB repair via homologous recombination (HR)^7^, replication fork protection^21^, and resolving stalled forks^22^. Thus, agents causing DNA damage and replication fork stress/stalling could be genotoxic for BRCA1 carriers due to the deficiency in the repair of stalled replication forks^23^ and elevated error-prone DNA repair in haploinsufficient BRCA1 cells^24^.

The mechanism leading to genomic instability in ER-negative haploinsufficient BRCA1 cells is not known. To investigate if estrogen and estrogen-inducing agents stall the DNA replication and induce genomic instability that results in cancer-initiating mutations, we examined ER-negative mammary cells carrying a BRCA1 germline mutation along with isogenic control cells. BRCA1^mut/+^ cells are haploinsufficient and have a mutation in one of the BRCA1 alleles. We found that estrogen (β-estradiol) and estrogen metabolites induce replication stress in BRCA1^mut/+^ cells. In addition, estrogen prompts DNA breaks and genomic instability, including deletions of the *BRCA1* gene (LOH). Surprisingly, we also detected loss of the entire chromosome 17 in BRCA1^mut/+^ cells exposed to estrogen, which is one of the characteristics of triple-negative breast cancer (TNBC) in BRCA carriers. Furthermore, we tested environmental pollutants and found that Atrazine, a known aromatase-activating agent, stalls the replication forks and induces DNA damage in BRCA1^mut/+^ cells. These findings reveal that cancer-inducing mutations in BRCA1^mut/+^ cells are prompted by estrogen metabolites and estrogen-inducing agents, which can be found endogenously or in the environment. To reduce DNA damage and genomic instability in BRCA1^mut/+^ cells exposed to estrogen, we tested several compounds. We identified that the dietary compound I3C prevents replication stress and reduces DNA damage in these cells. Thus, these results propose that I3C can reduce estrogen-induced mutagenesis in BRCA1 carriers and could be used as a chemopreventive option for ER-negative cancers.

## Results

### Estrogen and estrogen metabolites induce replication stress in BRCA1^mut/+^ mammary cells

**Fig. 1:**
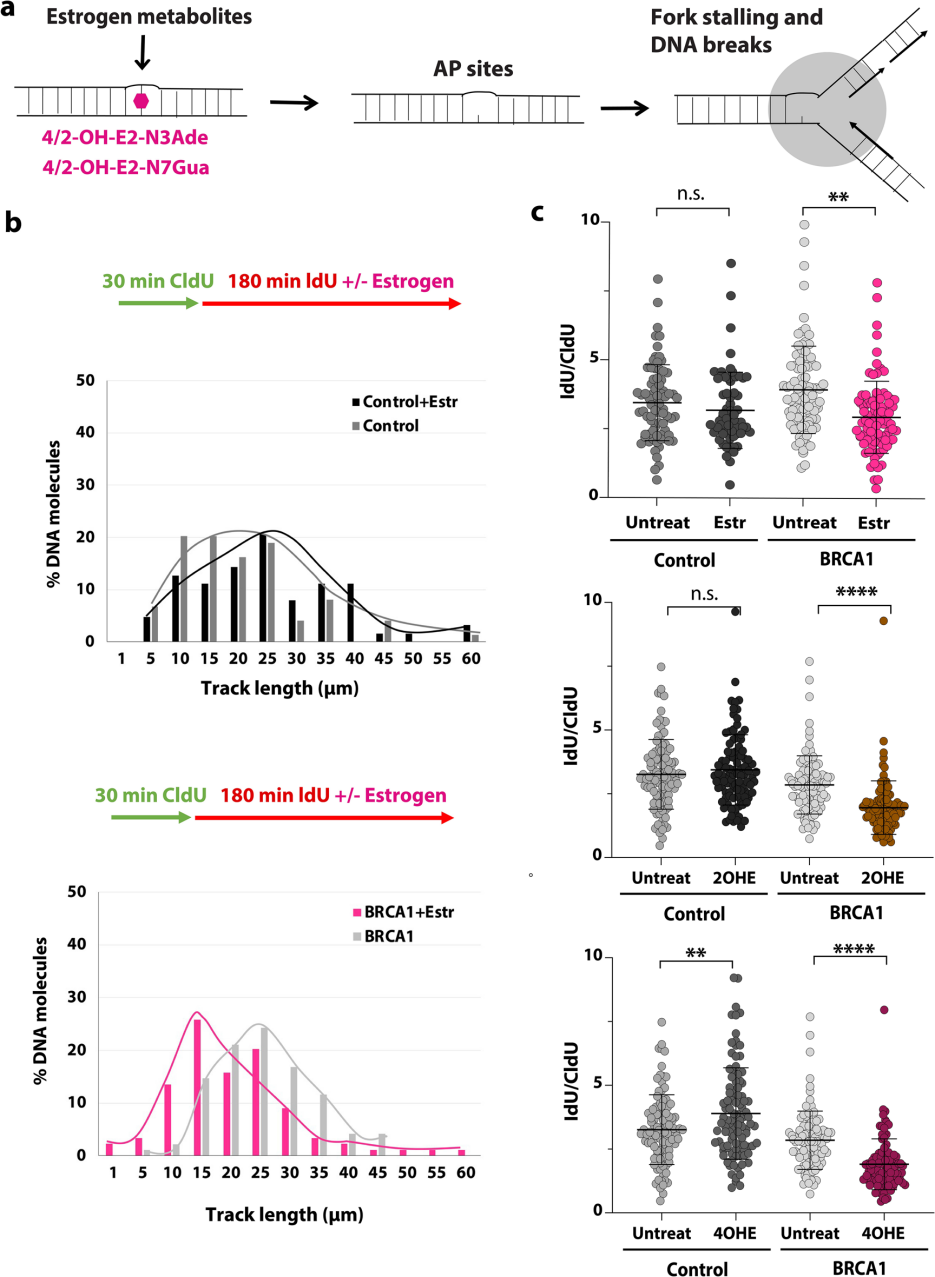
β-estradiol and estrogen metabolites stall the replication in ER-negative BRCA1^mut/+^ breast cells. **(a**) Illustration depicting how the formation of depurinating DNA adducts by estrogen metabolites leads to AP sites that can stall the fork and cause DNA breaks. **(b-c)** DNA fiber analysis of β-estradiol (Estr) (**b**) and estrogen metabolites (**c**) treated isogenic control and BRCA1^mut/+^ cells (185delAG mutation). **(b)** The percentage of DNA molecules with specific track length (µm) (second pulse, IdU) was calculated on the bottom, *n* = 80–100, *p*= *<0.05, ** <0.005. **(c)** Analysis of the fork rate (IdU/CldU ratio) is shown. The *p*-values and error bars are indicated. Statistical analysis was conducted two-sided Welch’s t-test for *p*-value calculation.

Most breast cancers that occur in women carrying a germline BRCA1 mutation are ER-negative^14^, and ER-negative breast cells are indicated to be the origin of TNBC^15^. Reduction of the endogenous estrogen levels by oophorectomy in *Brca1*-mutant mice results in a much lower ER-negative tumor incidence^25^, indicating that estrogen might have a role in cancer initiation in ER-negative mammary cells. However, the exact mechanism causing genomic instability and mutagenesis in ER-negative cells is not known. Estrogen is known to form depurinating DNA adducts. 17β-estradiol (E2) is oxidized by cytochromes to catechol estrogens (CEs; 2OHE & 4OHE). Then, CEs are oxidized by cytochrome P450s (CYPs) or peroxidases to highly reactive catechol estrogen quinones (E1/2–2,3-quinone and E1/2–3,4-quinone), which form DNA adducts (Fig. 1a). 4OHE generates transient DNA adducts within the genome that are proposed to be responsible for estrogen-induced mutations and carcinogenesis. Depurinating DNA adducts are quickly converted to apurinic (AP) sites, which could stall the DNA replication^26^.

**Fig. 2:**
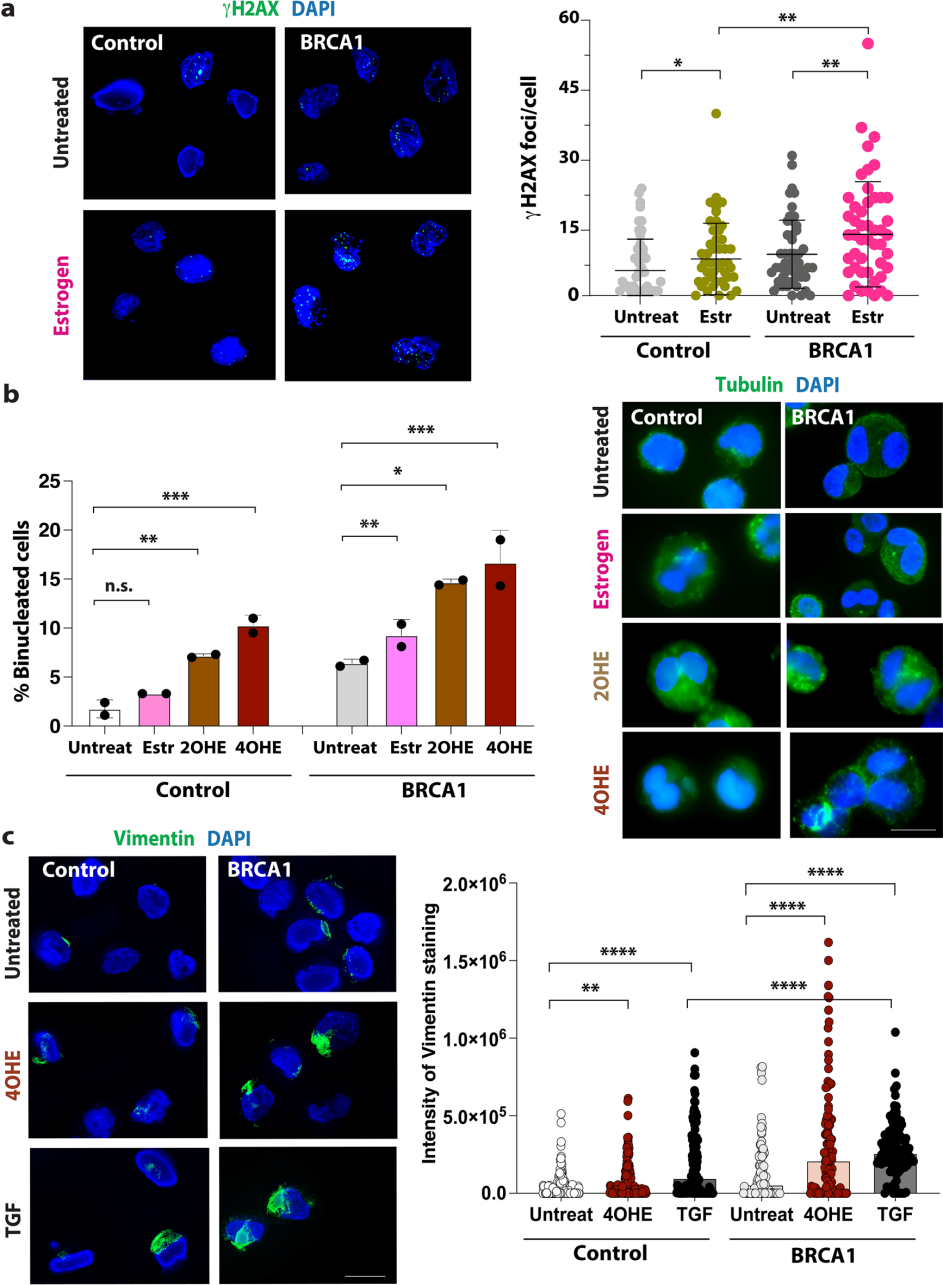
β-estradiol induces DNA breaks and cancer-like features in ER-negative BRCA1^mut/+^ breast cells. **(a)** Analysis of DNA breaks upon estrogen exposure. The number of γH2AX foci per nucleus (DAPI staining) was calculated. The *p*-values and error bars are indicated, *n* = 100, *p*= *<0.05, **<0.005, *** <0.0005. Representative images shown on left. **(b)** Analysis of binucleated cells in control and BRCA1^mut/+^ cells. Cells were treated with 1 µM β-estradiol (Estr) or estrogen metabolites for 72 h. The percentage of cells with two or more nuclei (*n* > 250) was calculated. Comparisons were made between treatments, and *p*-values were calculated and are indicated. Representative images are shown on the right. **(c)** Analysis of vimentin, a marker of EMT, in control and BRCA1^mut/+^ cells. The cells were treated with 1 µM estrogen metabolite 4OHE and TGF-**β** (positive inducer of EMT) for 72 h. Intensity of the vimentin staining was measured (*n* = 200, *p*-values < 0.001). Representative images are shown on the left. Statistical analysis was conducted using the Mann-Whitney U test for *p*-value calculation.

To determine if estrogen and estrogen metabolites affect the progression of the DNA replication in heterozygous ER-negative BRCA1 cells, we treated isogenic control and BRCA1^mut/+^ cells (MCF10A and hTERT-IMEC cells containing the 185delAG mutation^27^, referred to in the figure as BRCA1) with β-estradiol (also known as 17β-estradiol) and estrogen metabolites and performed DNA fiber experiments (Fig. 1b-c and Suppl. Figure 1b). Patients are exposed to β-estradiol and its metabolites throughout their lives (months or years). Since in vitro studies cannot be conducted for such a long time, most studies on cell lines use a concentration of around 1 to 300 µM of estrogen and estrogen metabolites^28,29^. Estrogen-DNA adducts are observed at this concentration (30 µM) in human mammary cell lines^28^. We pulse labeled the cells first with CIdU for 30 min, followed by ldU labeling with 1µM β-estradiol (Estr), 2OHE or 4OHE for 3 h (Fig. 1b-c). We analyzed two BRCA1^mut/+^ cell lines (BRCA1a has a R71G mutation in the RING domain of the *BRCA1* gene similar to 185delAG mutation). We detected shorter tracks of incorporated nucleotides (Fig. 1b ) and a reduced IdU/CldU ratio (Fig. 1c and Suppl. Figure 1a-b) in BRCA1^mut/+^ cells after treatment with β-estradiol and estrogen metabolites. Shortened IdU track and a reduced IdU/CldU ratio show that the replication fork progression was slowed down or stalled in these cells. We observed that replication fork progression was not hindered in isogenic control cells treated with β-estradiol and 2OHE. Thus, these results show that exposure to β-estradiol and estrogen metabolites, in particular 4OHE, caused replication stress in BRCA1^mut/+^ mammary cells. Induction of replication stress makes genomic DNA more vulnerable to DNA breaks and mutations, especially at difficult-to-replicate regions such as fragile sites. Concurrent with earlier findings^4^, we detected a significant increase in DNA breaks in BRCA1^mut/+^ cells using γH2AX staining (Fig. 2a) and alkaline comet assay (Fig. 5d) in cells that were exposed to β-estradiol.

**Fig. 3:**
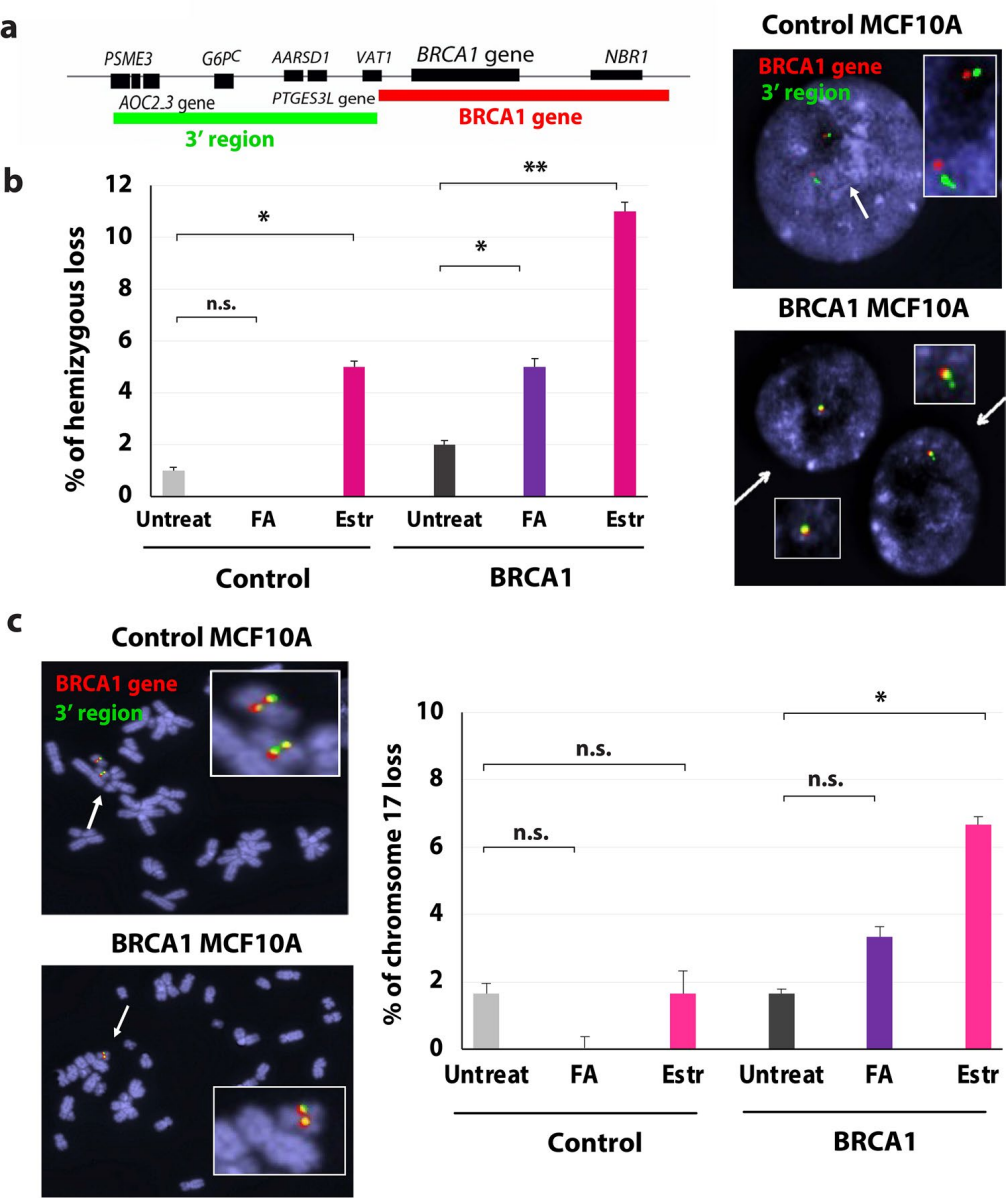
β-estradiol triggers LOH and deletion of chromosome 17 in ER-negative BRCA1^mut/+^ breast cells. **(a-c)** Analysis of metaphase spreads with FISH probes detecting the *BRCA1* gene and 3’ control regions **(a)** Map of the FISH probe binding sites, **(b)** Percentage of isogenic control and BRCA1^mut/+^ cells with hemizygous loss after exposure to formaldehyde (FA) or β-estradiol (Estr), error bars are shown, *n* = 60. Representative image of interphase cells analyzed by FISH on right. **(c)** Percentage of isogenic control and BRCA1^mut/+^ cells with deletions of chromosome 17 exposed to FA or β-estradiol (Estr), error bars are shown, *n* = 60. Representative image of metaphase spreads and loss of chromosome 17 on left. Statistical analysis was conducted using Student’s *t*-test with two-tailed distribution for *p*-value calculation.

Since replication stress also arrests or prolongs the time cells are in S-phase, we performed PCNA/EdU assay on β-estradiol-treated cells. We treated isogenic control and BRCA1^mut/+^ cells with 1µM estradiol for 6 h (Suppl. Figure 1c). We added EdU to the last 30 min and performed Click-it Chemistry (using Click-It EdU Imaging kit, Invitrogen). Then, we quantified the number of cells with EdU and PCNA staining. As positive control, we treated cells with aphidicolin, an inhibitor of replication and DNA-polymerase alpha. As expected, aphidicolin treatment increased the number of EdU/PCNA cells. Like aphidicolin and in contrast to control, β-estradiol caused a slight increase in the number of EdU and PCNA-positive cells, which indicated that estrogen prolonged S-phase in BRCA1^mut/+^ cells upon exposure. In summary, these results suggest that β-estradiol and estrogen metabolites hinder the replication forks and induce DNA breaks in BRCA1^mut/+^ mammary cells.

### Estrogen induces genomic instability, deletions, and cancer-like features in ER-negative BRCA1^mut/+^ breast cells

To test if β-estradiol and estrogen metabolites induce genomic instability, we performed several assays. First, we examined the formation of micronuclei (MN). MN formation in mammalian cells serves as a marker of genomic instability and genotoxic stress^30^. It was reported that mild replication stress is sufficient to induce mis-segregation of entire or near-entire chromosomes in non-cancerous cells, leading to micronuclei (MN) formation^31^. MN are also formed by acentric chromosome fragments produced by unrepaired DSB breaks, for example, that were introduced during DNA replication^32^. To test if estrogen induces MN formation in BRCA1^mut/+^ cells were exposed to 1µM β-estradiol, and micronuclei were quantified (Suppl. Figure 2a). As a positive control, we exposed cells to a low concentration of aphidicolin. Only a few micronuclei were found in control and BRCA1^mut/+^ cells. However, the number of MN was increased in BRCA1^mut/+^ cell lines that were exposed to β-estradiol, in contrast to control cells (with and without treatment).

Surprisingly, we found an increase in binucleated BRCA1^mut/+^ cells (Fig. 2b**)**. Binucleation is an often-observed feature of cancer cells and a marker of genomic instability. Chromosome segregation during mitosis can be impaired when the DNA replication is incomplete or compromised due to stalled forks. If the cells attempt to divide despite incomplete DNA replication, this can result in cells containing two nuclei (binucleation). Various cells have elevated numbers of binucleated cells, including breast tumors^33,34^. We found that in BRCA1^mut/+^ cells, β-estradiol and estrogen metabolites produced a significant increase in the percentage of binucleated cells, but only in the BRCA1^mut/+^ cells containing the 185delAG mutation (BRCA1, Fig. 2b). BRCA1a^mut/+^ cells containing the R71G mutation have fewer binucleated cells and treatment seems to have no significant effect (Suppl. Figure 2b-c). It was previously reported that CIN (chromosomal instability) varies between the deleterious germline mutation and that BRCA1^mut/+^ cells with R71G mutation had a less severe phenotype as BRCA1^mut/+^ cells with the 185delAG mutation^35^. This could be because part of the RING domain is still expressed as a truncated protein in these cells, thus preserving some BRCA1 function.

Another common feature of cancer cells is an increase in vimentin, a key marker for epithelial-mesenchymal transition (EMT)^36^. To study whether treatment with β-estradiol or estrogen metabolites increases the number of cells with vimentin (Fig. 2c), we analyzed the vimentin expression in isogenic control and BRCA1^mut/+^ cells. As a positive control, we treated cells with TGF-β, a known factor that induces EMT^37^. Since the estrogen-metabolite 4OHE considerably induced replication stress and binucleation, we treated the BRCA1^mut/+^ cells and isogenic control cells with 4OHE. As expected, we detected a significant increase in vimentin expression in BRCA1^mut/+^ mammary cells that were treated with TGF-β in comparison to the control cell line. We also detected a significant increase in vimentin expression in BRCA1^mut/+^ cells that were treated with 4OHE. These results show that the exposure to 4OHE increases the percentage of cells with vimentin expression in ER-negative BRCA1^mut/+^ cells. In summary, these findings show that β-estradiol and its derivatives induce characteristics that are known features of carcinogenic BRCA1 cells.

Next, we tested whether estrogen induces large deletions and cancer-initiating mutations, such as deletions at the *BRCA1* gene and LOH in BRCA1^mut/+^ cells. The *BRCA1* gene is a fragile site that is prone to fork stalling, DNA breaks, and deletions^24^. In our earlier studies, we found that stalled forks are repaired by error-prone DNA repair, causing deletions at these sites in BRCA1^mut/+^ cells. To test if β-estradiol induces deletions at the *BRCA1* gene and LOH, we performed FISH on interphase chromosomes with two probes (one binding at the *BRCA1* gene and the other at 3’ control regions) (Fig. 3a). As control we treated isogenic control and BRCA1^mut/+^ cells with high concentration of formaldehyde (FA, 100 µM), previously reported to stall the replication fork and cause genomic alterations in BRCA2-deficient cells^38^. We also used a higher concentration of β-estradiol (30 µM), since we treated only for a short time in comparison to the estrogen exposure patients endure throughout their lives. The results show that estrogen prompted deletions of the *BRCA1* gene and LOH in BRCA1^mut/+^ cells (Fig. 3b). These results also reveal that both FA and β-estradiol are mutagenic, inducing LOH in BRCA1^mut/+^ cells. However, β-estradiol notably caused a significantly higher number of deletions and LOH, thus it is more mutagenic than 100 µM FA. In addition, we also examined metaphase chromosome spreads (Fig. 3c). Surprisingly, we detected an increase in loss of the entire chromosome 17 in BRCA1^mut/+^ mammary cells after exposure to β-estradiol. In contrast untreated cells and control cells did not lose chromosome 17. Deletion of chromosome 17 has been reported in numerous BRCA1 cancer cells^10^. In summary, these results show that β-estradiol induces genomic instability, cancer-like features, and initiates LOH in BRCA1^mut/+^ cells.

**Fig. 4:**
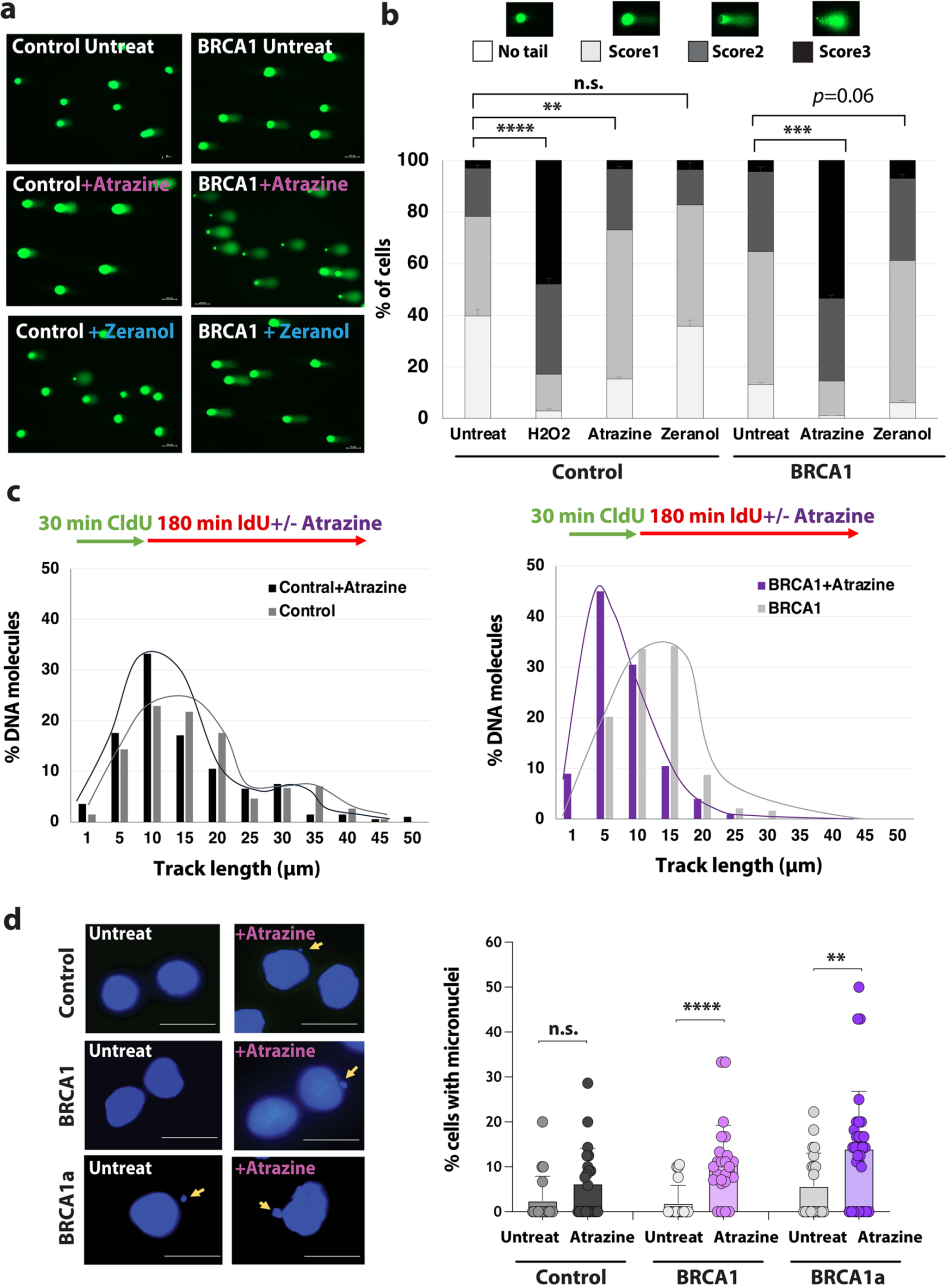
An environmental pollutant causes replication stress and genomic instability in BRCA1^mut/+^ cells. **(a-b)** Analysis of DNA breaks in control and BRCA1^mut/+^ cells using alkaline comet assay. Cells were treated with 1 µg/ml Atrazine or 10 ppb Zeranol for 8 h. The percentage of cells with comet tails (Score 1–3, *n* = 100), error bars, and *p*-value are indicated. Representative images are shown on the left. **(c)** DNA fiber analysis of 1 µg/ml Atrazine-treated control and BRCA1^mut/+^ cells. The percentage of DNA molecules with specific track length (µm) (second pulse, IdU) was calculated, *n* = 100. **(d)** Analysis of the genomic instability in control and BRCA1^mut/+^ cells treated with and without 2 µg/ml Atrazine (Atraz) using micronuclei assay. Representative images are shown on the left. The percentage of cells with MN (*n* > 100) and *p*-values are shown. Statistical analysis was conducted using two-sided Welch’s t-test for *p*-value calculation. *p*-values are indicated: * <0.05 ** <0.005 *** <0.0005, **** <0.00005.

### An estrogen-inducing environmental pollutant inhibits replication fork progression and promotes genomic instability in ER-negative BRCA1^mut/+^ breast cells

Numerous environmental pollutants that cause mammary gland tumors in animals are shown to be hormonally active and mimic or induce estrogen production^39^. Because of only minimal insights and studies on the effect of these chemicals and their metabolites on humans, few of these compounds have been considered so far as potentially harmful. Environmental pollutants with estrogenic activity or that elevate estrogen production could have a major impact on the health and cancer initiation in BRCA1 carriers. The genotoxic potential of estrogen-inducing pollutants, like Zeranol and Atrazine (Suppl. Figure 3a), has not been examined in repair-deficient cells, such as BRCA1 carrier cells. Atrazine is an herbicide and is used to prevent pre-emergence broadleaf weeds in crops such as maize and sugarcane. Zeranol, on the other hand, is a growth promoter commonly used in livestock, particularly beef cattle. It was also reported that Zeranol and Atrazine stimulate cell proliferation and aromatase activity^40,41^. Excessive use and runoff from agricultural areas can contaminate drinking water and cause health problems. A study showed that Atrazine was the most commonly detected pesticide contaminating drinking water in the US in 2001^42^.

A previous study indicates that Atrazine induces the formation of γH2AX foci, thus, DNA breaks in MCF10A cells^43^. To determine if Atrazine and Zeranol prompt DNA breaks in BRCA1^mut/+^ cells, we analyzed these cells using the alkaline comet assay. As a positive control, we exposed cells to Hydrogen peroxide (H_2_O_2_), which is known to be genotoxic. We found that Atrazine increased the number of DNA breaks in BRCA1^mut/+^ cells (Fig. 4a-b). Between the two environmental factors, Atrazine but not Zeranol induces DNA breaks in the genomic DNA in BRCA1^mut/+^ cells. We used a concentration of Atrazine (1-2 µg/ml), which is not cytotoxic in control and BRCA1^mut/+^ cells (Suppl. Figure 3b-c). Since Atrazine induced a high number of DNA breaks, we tested whether this environmental pollutant inhibits DNA replication. Therefore, we performed DNA fiber analysis. We treated control and BRCA1^mut/+^ cells with Atrazine (1 µg/ml) during the second pulse (Fig. 4c). We found shorter IdU tracks in Atrazine-exposed BRCA1^mut/+^ cells (Fig. 4c and Suppl. Figure 3 d), indicating that Atrazine inhibits replication fork progression in BRCA1^mut/+^ cells. Next, using the MN assay, we determined if Atrazine prompts genomic instability. We detected increased MN formation after exposure of BRCA1^mut/+^ cells with 2 µg/ml Atrazine (Fig. 4d). Treatment with Atrazine also induced a significant increase in the percentage of binucleated BRCA1^mut/+^ cells (Suppl. Figure 3e). In summary, Atrazine induces replication stress and DNA breaks that can cause genomic instability in BRCA1^mut/+^ cells.

**Fig. 5:**
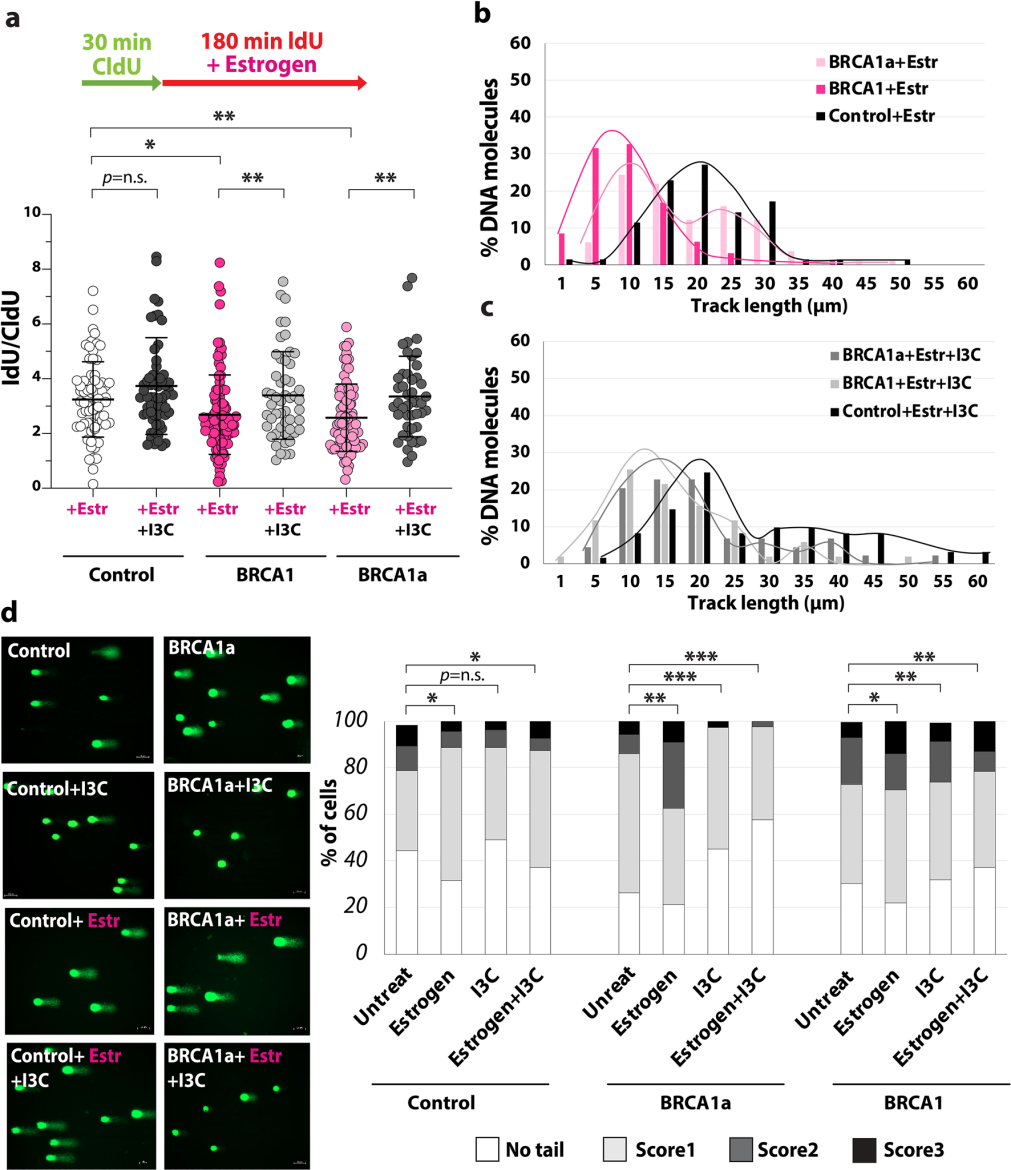
Dietary compound I3C impairs replication fork stress and reduces DNA damage in BRCA1^mut/+^ cells. **(a**-**c)** DNA fiber analysis of estrogen-treated control and BRCA1^mut/+^ cells with and without 100 µM I3C. **(a)** Experiment procedures are shown on **top in c** and the fork rate (IdU/CldU ratio) on the bottom. The *p*-values and error bars are indicated, *n* = 50–100, *p*=*<0.05, ** <0.005. ANOVA: F = 6.7 *p* = < 0.0001. **(b-c)** The percentage of molecules with track lengths between 1–60 μm (second pulse, IdU) is shown for β-Estradiol (Estr) (**b**) and β-estradiol/I3C-treated cells (Estr+I3C) (**c**). **(d)** Analysis of the DNA damage in control and BRCA1^mut/+^ cells treated either with or without 1µM β-estradiol (treated for 8 h) and/or 100 µM I3C using the comet assay. Representative images are shown on left. The percentage of cells with no tail, score 1, score 2 and score 3 was quantified (*n* = 120). *p*-values are indicated: * <0.05 ** <0.01 *** <0.001, **** <0.0001. Statistical analysis was conducted using one-way ANOVA followed by Brown–Forsythe and Welch tests for *p*-value calculation.

### Indole-3-carbinol prevents replication stress and DNA damage in BRCA1^mut/+^cells

Next, we tested whether the dietary compound Indole-3-carbinol (I3C), which was associated with reduced breast cancer risk in epidemiological studies and animal models^44^, can prevent estrogen-induced replication stress (Fig. 5a-c). It was shown that I3C induces BRCA1 expression in cancer cells, such as MCF7^45^. We wondered if an increase in BRCA1 expression after I3C treatment could prevent replication stress and DNA breaks. As previously described, I3C increased the BRCA1 expression in BRCA1^mut/+^ cells. (Suppl. Figure 4a-b). Next, we analyzed the replication fork progression using the DNA fiber assay. We found that a low concentration of I3C (100 µM) was able to avert β-estradiol-induced replication stress in isogenic BRCA1^mut/+^ cells (Fig. 5a-c and Suppl. Figure 4c). In both cell lines, the IdU tracks were significantly longer than in β-estradiol-only treated cells. I3C also released the replication stress in BRCA1^mut/+^ cells that were exposed to Atrazine (Suppl. Figure 4e). Next, we examined whether I3C can reduce the number of DNA breaks in β-estradiol-treated BRCA1^mut/+^ cells. Using the comet assay, we found that in both BRCA1^mut/+^ cells treated with I3C the number of DNA breaks was considerably reduced in β-estradiol-treated BRCA1^mut/+^ cells (Fig. 5d and Suppl. Figure 4 d). In summary, these results show that I3C can counteract β-estradiol-induced DNA damage in BRCA1^mut/+^ cells by reducing replication defects in these cells. These results also demonstrate that compounds that can avert replication stress can prevent DNA breaks in heterozygous BRCA1 ^mut/+^ cells. Thus, we were able to identify a new natural compound that can avert replication stress and DNA breaks by restoring the BRCA1 expression in BRCA1^mut/+^ mammary cells.

## Discussion

Endogenous and environmental factors that induce cancer development in ER-negative breast cells are not known. It was discovered that BRCA1 triple-negative cancer cells originate from ER-negative breast progenitor cells^15^. MCF10A breast cells express luminal, stem, and progenitor cell markers^46,47^; thus is one of the most suitable cell lines to analyze the mechanisms leading to cancer initiation in BRCA1 carriers. It is known that estrogen induces DNA damage by binding to the ER and inducing R-loops (ER-dependent mechanism). However, our study demonstrates that estrogen, estrogen metabolites, and an endocrine-disrupting pollutant are able to induce replication stress (Figs. 1 and 4) and genomic instability (Figs. 2 and 3) in ER-negative breast cells (ER-independent mechanism), particularly in BRCA1^mut/+^ cells that are deficient in the repair of stalled forks. Due to only 50% of BRCA1 expression in BRCA1^mut/+^ cells^24^, the stalled forks cannot be fixed properly, causing breaks, genomic instability, such as micronuclei formation and deletions in these cells. However, the deletions induced by estrogen are larger as compared to other fork stress/stalling agents^24^ and the deletion also include 3’ adjacent regions of *BRCA1* gene (Fig. 3). This could be explained by the formation of estrogen-inducing DNA adducts that affect more regions in the cells and not just fragile sites (*BRCA* genes^24^, but also repetitive DNA sequences outside these regions.

DNA damage can prime the genomically unstable heterozygous BRCA1^mut/+^ cells to induce cancer-like features. Indeed, we observed genomic instability, deletions, and an increase in vimentin, an EMT marker (Fig. 2). EMT is an important process in tumor progression, and it is also a classical feature of cancer. Activation of EMT by estrogen has been reported in double-mutant mammary tumors with p16 and p18 deficiency, suggesting that estrogen induces EMT in BRCA1-deficient tumors, which are dependent on AKT activation, but independent of ER^48^. However, such studies are limited to animal models and cancerous cell lines. Other characteristics of cancer cells also include cell cycle dysregulation, binucleation and LOH (for BRCA carriers loss of the second BRCA allele)^9,33,34,49^. Our results demonstrate that exposure to estrogen induces cancer-initiating mutations (LOH) and carcinogenic features in ER-negative BRCA1^mut/+^ cells (Figs. 2 and 3). Studies conducted so far suggest that defects during repair of DNA adducts by Base Excision Repair (BER) or Nucleotide Excision Repair (NER) lead to mutations, such as single-nucleotide mutations. However, these defects would not cause large deletions, which are observed in BRCA1 cancer cells. Our results show that estrogen exposure leads to larger mutations, such as deletions of the *BRCA1* gene and adjacent regions. Even an increase in loss of the whole chromosome 17 was observed in estrogen-treated BRCA1^mut/+^ cells. These results align with the LOH observed in cancer cells in BRCA1 patients, where deletions of large parts and sometimes the entire loss of chromosome 17 (which also contains the *p53* gene) occurred^9,50^. These DSB are probably caused by error-prone repair of the stalled forks, such as MMBIR, which we detected previously to be elevated after induction of replicative stress in BRCA1^mut/+^ cells. We were able to prevent DNA breaks and replication fork stress by treatment with I3C, a natural compound and nutritional supplement. I3C and its metabolites were previously reported to have cancer-preventive activities^44,51^.

Estrogen is necessary for the normal development and growth of the mammary gland, control of the woman’s menstrual cycles, and is necessary for reproduction. However, prolonged exposure to estrogen has been associated with an increased risk of breast cancer^52^. The risk of developing breast cancer is increased in pre-and post-menopausal BRCA carriers who had early onset of menarche and late menopause^53,54^, probably due to exposure to higher unbalanced levels of estrogen. An increase in estrogen levels can occur due to unbalanced hormone levels, environmental pollutants, or obesity^12^. Reduction of serum estrogen levels by oophorectomy protects against breast cancer shown in a mouse model^25^ and BRCA1 patients^55–57^. In ER-positive MCF7 cells, estrogen induces DNA damage by binding to ER and activating the transcription that leads to increased R-loop formation^13,58^. Estrogen also induces proliferation and reduces apoptosis in ER-positive cells, thus enhancing cancer development (reviewed in^59^). This led to the thinking that estrogen is mostly a transcriptional or epigenetic carcinogen. However, reducing estrogen by oophorectomy in high-risk carriers of BRCA1 mutations greatly reduces the overall incidence of invasive ER-negative breast cancer^60^. Our results reveal that estrogen and estrogen metabolites are genotoxic for DNA repair-deficient ER-negative breast cells in contrast to ER-positive breast cells, where estrogen is mostly shown to cause transcription defects and epigenetic toxicity.

We also found that the environmental pollutant Atrazine caused replication fork stress and DNA damage in ER-negative BRCA1^mut/+^ cells. The safety of Atrazine remains controversial^61^. Atrazine is banned in other countries in the world due to its toxicity. However, in the US, Atrazine is still approved and one of the most commonly found herbicides in tap water. Atrazine increases aromatase levels by inhibiting phosphodiesterase^3,62^, resulting in elevated cAMP in human cancer cell lines. Elevated cAMP results in increased transcription of the aromatase gene CYP19 (cytochrome P450, family19) and increased aromatase activity, leading to increased estrogen production^63^. We found that Atrazine significantly stalled the replication fork, induced DNA breaks, and genomic instability in ER-negative BRCA1^mut/+^ cells (Fig. 4). Atrazine is an activator of the aromatase and estrogen production. We were able to prevent β-estradiol- and Atrazine-induced DNA damage by treatment with the dietary compound I3C (Fig. 5, and see model Suppl. Figure 5). These results show that it is important to understand the specific environmental and endogenous factors and mechanisms that promote cancer development to identify compounds that minimize the cancer risk in DNA repair-deficient carriers, such as BRCA1 carriers, and prevent cancer initiation and progression in these patients.

## Limitations

Our results explain how estrogen and estrogen metabolites lead to larger mutations, specifically deletions of the *BRCA1* gene and surrounding regions in ER-negative BRCA1 carrier cells.

However, besides deletions also other mutations, such as single-nucleotide mutations, were observed in BRCA1-deficient cells^64^ and BRCA1 patients^10^. Thus, other cellular pathways and factors, such as translesion polymerases, and defects during repair of AP sites, probably by base excision repair (BER), in addition, could contribute to the wide range of mutations in BRCA1 cancer cells. In addition, numerous environmental pollutants that cause mammary gland tumors in animals are shown to be hormonally active and mimic or induce estrogen production^39^. Testing of additional environmental agents and cell lines carrying the BRCA1 (185delAG) mutation or other BRCA germline mutations will broaden the findings. The current research focuses exclusively on heterozygous BRCA1 breast epithelial cells. Testing the genotoxic effects of estrogen on other tissues would be interesting, but it is outside the scope of the study. Also, we used a higher concentration of β-estradiol and Atrazine than are found physiologically^65,66^, to compensate for the limitations of in vitro studies in mimicking long exposure times. Additionally, Atrazine and I3C are known to induce broad metabolic and proliferative changes, which may also indirectly alter replication track lengths, and further experiments are needed to exclude other mechanisms. Thus, there is a need for further evaluation of estrogen and environmental factors to understand which factors and agents introduce mutations and elevate the risk of cancer development in BRCA1 carriers with various germline mutations.

### Experimental model and subject details

#### Cell lines

 We used MCF10A (a non-tumorigenic, immortalized human mammary epithelial cell line) alongside hTERT-IMEC (TERT immortalized human mammary epithelial cells) in the present study. The haploinsufficient BRCA1 mammary cells, contain one allele with a founder mutation (185delAG) and an unaffected sequence on the other allele (referred to in the figures as BRCA1). The BRCA1^mut/+^ hTERT-IMEC also contains the 185del AG mutation. In addition, we used a second haploinsufficient BRCA1 MCF10A cell line (referred to as BRCA1a) with a R71G mutation in one allele (a Arg to Gly change at codon 71). The haploinsufficient BRCA1 breast cell lines were generated in the same isogenic control MCF10A and hTERT-IMEC cell lines^27^, and were a gift from Dr. Ben Ho Park (Vanderbilt University). We also obtained control MCF10A cells from Dr. Nagarajan Kannan’s lab (Mayo Clinic).

#### Cell culture

The human control and haploinsufficient MCF10A BRCA1 breast cells were grown in DMEM/F12 media (Sigma Aldrich, #11330-032) supplemented with 5% horse serum (ThermoFisher Scientific, #16050-122), 20 ng/mL epidermal growth factor (Peprotech, #AF-100-15), 10 µg/mL insulin (Sigma Aldrich, #I-1882), 0.5 µg/mL hydrocortisone (Sigma Aldrich, #H088), 0.1 µg/mL cholera toxin (Sigma Aldrich, #C-8052) and 1% Penicillin-Streptomycin (ThermoFisher Scientific, #15070063). The cells were passaged every 3–5 days by using TrypLE (ThermoFisher Scientific, #12604013). Cells were maintained in a 37 °C incubator with 5% CO2.

The human hTERT-IMEC control and haploinsufficient BRCA1 breast cells were grown in MEGM Mammary Epithelial Cell Growth Medium (Lonza #CC-3150). The cells were passaged every 4–5 days by using TrypLE (ThermoFisher Scientific, #12604013). Cells were maintained in a 37 °C incubator with 5% CO2.

### DNA fiber analysis

As we previously described^24^, cells were pulsed-labeled with 25 µM 5-chloro-2′-deoxyuridine (CldU) (MP Biomedicals, #ICN10547883) for 30 min and followed by incubation with 250 µM 5-iodo-2′-deoxyuridine (IdU) (MP Biomedicals, #ICN10035701) for 3 h. Cells were treated during second pulse with and/or 5 µM PARPi (Olaparib AZD2281) (Selleckchem, #S1060), 1 µM Estrogen (β-Estradiol) (Sigma Aldrich, #E2758), 1 µM 2OHE (Cayman Chemical, # 362-05-0), 1 µM 4OHE (Sigma Aldrich, # H4637),100 µM I3C (Sigma Aldrich #I7256), 1ug/ml Atrazine (Sigma #49085) during the second pulse for 3 h to assess effect on replication. Untreated cells were used as controls. DNA fiber assay was performed as previously described^67^. Briefly, cells were sequentially pulse labeled with 25 µM CldU followed by 250 µM IdU for 30 min at 37℃. Cells were washed with ice-cold PBS, trypsinized, and resuspended at 2.5 × 10^6^ cells/mL concentration in cold PBS. 2 µl of cell suspension was gently placed on glass slide and after incubation of 2–3 min, lysis buffer (200 mM Tris-HCl; pH 7.5, 50 mM EDTA and 0.5% SDS) was added, followed by 2 min of incubation at room temperature. The slides were tilted at an angle (15–45°) to allow the fibers to spread along the slide. After drying, the slides were fixed in methanol-acetic acid (3:1) for 15 min at −20℃, denatured in 2.5 M HCl for 30 min, and blocked in 5% BSA in 1X PBS for 20 min (blocking buffer). Primary anti-BrdU antibodies specific for CldU (1:15, Abcam, #ab6326) and IdU (1:15, Becton Dickinson; cat # 347580) were applied for 1 h at room temperature followed by PBS wash. Slides were then stained with secondary antibodies; anti–rat Alexa Fluor 488 (1:15, Invitrogen, #A-11006) and anti–mouse Alexa Fluor 568 (1:15, Invitrogen, #A-11031) for 1 h at room temperature. Slides were mounted with ProLong Antifade Mountant (Thermo Fisher Scientific, #P10144,) and imaged on Carl Zeiss Axio Imager M2 microscope. Labeled replication structures were identified, and the track length was measured using ImageJ. The fork rate was analyzed by calculating the IdU/CldU ratio. We used two-sided Welch’s t-test to assess the statistical significance for *p*-value calculation.

### Comet assay

Cells were incubated with 1 µM Estrogen (β-Estradiol) or PARPi (Olaparib AZD2281) or 100 µM I3C (Indole-3-carbinol,Sigma Aldrich #I7256) along with Aphidicolin (Sigma Aldrich, #A0781) or Estrogen , or Atrazine (1 µg/ml; Milipore Sigma, #49085) or Zeranol (10 ppm; Sigma Aldrich, #Z0292) for 8 h or Hydrogen peroxide (100 µM for 5 min, Sigma Aldrich, #H1009). Untreated cells were used as controls. Alkaline comet assays were performed according to^68^ with slight modifications. In brief, the cells were harvested, washed with ice-cold PBS and cell count was adjusted to 1 × 10^5^ cells/ml. The cells were mixed with molten Low melting agarose (1% prepared in 1X PBS), then 75 µl (500–1,000 cells) was immediately added to the comet slide (pre-coated agarose slides-1%). The slides after gelling were incubated in lysis buffer (2.5 M NaCl, 100mM EDTA pH 8, 10mM Tris HCl and 1%Triton X; pH adjusted to 10) for 2 h at 4 °C. The slides were washed twice with incubated in alkaline electrophoresis solution (300mM NaOH, 1mM EDTA, pH > 13, freshly prepared and stored at 4 °C) and incubated in same buffer for 30 min. Electrophoresis was performed at 30 V (1 V/cm- length between two electrodes) and 300 mA for 25 min. The slides were rinsed with neutralization buffer (400mM Tris HCl pH 7.5) and placed in increasing concentration of ethanol (70, 90 and 100%) ethanol for 5 min and then air-dried. YOYO-1 staining was done to visualize the DNA, wherein 150 µl of a 2µM of YOYO-1 (ThermoFisher Scientific, #Y3601) in TE was added to each slide and incubated for 15 min. The slides were dried and mounted. The comets were visually scored using fluorescence microscopy (Carl Zeiss Axio Imager M2 microscope). Comets were scored based on the presence of a tail and intensity of tail. A visual scoring method that classifies comets from grades 0–3 was used, where in the score 0 represented undamaged cells (comets with no or barely detectable tails); score 1- 5–30% of migrated DNA; score 2- 31–70% of migrated DNA and score 3- >70% of migrated DNA. The % comets per treatment as well as % scores were calculated from 2 independent experiments. Statistical significance was determined using one-way ANOVA followed by Brown–Forsythe and Welch tests.

### Slide preparation and Immunofluorescence staining

As we previously described^24,69^, harvested cells were gently placed on poly-lysine coated slides (Shandon, #6776216). The cells were allowed to settle for 20 min at RT. Liquid was removed by gently tilting the slide and the cells were fixed permeabilized in PMTEF buffer for 20 min at RT (4% paraformaldehyde, 200mM PIPES-pH 6.8, 200mM MgCl_2_, 10mM EGTA, 0.2% Triton X). After fixation-permeabilization, slides were washed three times with PBS. The cells were blocked with 1%BSA and 0.3% Triton X in PBS and then incubated overnight with primary antibody at 4 °C. This was followed by two PBS washes and incubation with appropriate secondary antibody (1:1000, Alexa Fluor 488 Cell Signaling Technology, #4412) for 1 h at room temperature. Primary antibodies used were Phospho-Histone H2A.X (1:1000, Cell signaling technology, # 9718 T), Tubulin Alpha (1:100, BioRad, # MCA77G), Vimentin (1:100, Cell Signaling Technology, # 5741 T). The slides were mounted with ProLong Gold Antifade Mountant with DAPI. A fluorescent microscope (Axioscop 2 M2 with Plan Apochromat 63×/1.4 NA oil differential interference contrast objective; Carl Zeiss) with a camera (CoolSNAP HQ; Photometrics) was used for imaging. For qualitative analysis, single plane images were taken using a fluorescent microscope. ≥100 cells were scored to calculate % cells with positive staining from 2 independent experiments.

For foci counting, Z-stacks of images were collected (0.25 μm) and were subjected to constrained iterative deconvolution. 2D-images were generated using maximum projection. The number of foci per nucleus was calculated by individually counting the number of foci present in each cell nucleus using ImageJ software. Average ± Std Deviation was calculated from 2 independent experiments and ≥ 100 cells were scored for each experiment. Statistical analysis was conducted using two-sided Welch’s t-test for *p*-value calculation. For fluorescence intensity, the level of cellular fluorescence was determined from the images using ImageJ software. The Median with 95% CI was calculated from 2 independent experiments and ≥ 150 cells were scored for each experiment. Statistical analysis was conducted using the Mann-Whitney U test to compare the differences among groups.

### PCNA/EdU

The cells were treated with 1 µM Estrogen or 0.8 µM Aphidicolin for 6 h. Equivalent amount of Ethanol was used as control. The cells were treated with EdU (10 µM, Invitrogen, A10044) for 30 min, before harvesting. After harvesting, polylysine slides were prepared, and immunostaining was done as described earlier^24,70^. Slides were incubated overnight with PCNA primary antibody (1:1000, Anti-PCNA rabbit polyclonal, Abcam #ab18197) at 4 °C followed by incubation with secondary antibody (Goat anti-rabbit Alexa Fluor 594, 1:1000, Invitrogen, #A-11005;) for 1 h at room temperature. The cells were washed with PBS thrice before proceeding for Click-it Chemistry (Invitrogen, #C10337). The reagents were prepared according to the manufacturer’s instructions. Cells were incubated with permeabilization buffer followed by incubation with Click-it cocktail for 30 min. The slide was washed thrice with PBS and mounted with ProLong Gold Antifade Mountant with DAPI (ThermoFisher Scientific, #P36935). Statistical analysis was conducted using a two-sided Welch’s t-test for *p*-value calculation.

### Micronuclei assay

For the micronuclei assay (also see^70^, cells were fixed on polylysine slides as mentioned earlier. For EdU and Micronuclei co-localization assay, cells were treated with EdU (10 µM, Invitrogen, A10044) for 30 min prior to harvesting, and Click-it chemistry was performed as described above and slides were mounted with Antifade Mountant with DAPI. The slides were allowed to dry before scoring for micronuclei. The slides were read on Zeiss fluorescence microscopes at 100X magnification. The nuclei were assessed for the presence of micronuclei, and micronuclei bridges. Average ± Std Deviation was calculated from 3 independent experiments and ≥ 120 cells were scored for each experiment. We used a two-sided Welch’s t-test for *p*-value calculation.

### Cell viability assay

As we previously described^24^, to determine the effect of atrazine on cell viability, the cells were treated with different concentrations of Atrazine (dissolved in DMSO). Briefly, the cells were seeded at a density of 10,000 cells/well in 96-well plates and cultured overnight. The cells were then treated with atrazine at various concentrations (0.5, 1, and 5 µg/ml) for 24 h. Control cells were incubated with an equivalent amount of DMSO. After incubation, the cell viability was assessed using CCK-8 kit (Abcam, #ab228554). WST-8 reagent provided was added to each well, and plates were incubated for 4 h at 37 °C. The plates were gently mixed on an orbital shaker, and then the absorbance of each sample was measured at 460 nm using a Tecan microplate reader. Percent cell viability was calculated according to the manufacturer’s instructions.

### Semi-quantitative RT-PCR analysis

As we previously described^24^, mRNA expression was determined by semiquantitative RT–PCR assays. Briefly, the cells were treated with 100µM I3C for 8,16, and 24 h. Untreated cells were used as control. The cells were harvested, and RNA extraction was performed using PicoPure RNA Isolation Kit (Applied Biosystems, # KIT0204). cDNA synthesis was done by using iScript Reverse Transcription Supermix (Biorad, #1708840). This was followed by PCR using specific primers for BRCA1 and beta-actin (control gene). The details of the primers and PCR reaction have been described before (BRCA1 Forward primer TTGCGGGAGGAAAATGGGTAGTTA, BRCA1 Reverse primer TGTGCCAAGGGTGAATGATGAAG, Actin Forward primer TGTTACCAACTGGGACGATA, Actin Reverse primer GATCTTGATCTTGGTGCT). The PCR products were analyzed by electrophoresis through 1% agarose (Seakem, #50070) gels with 0.1 mg/ml of ethidium bromide and gels were photographed under ultraviolet light. The ratio of BRCA1 expressed as compared to actin was quantified using ImageJ software.

### Karyotyping and FISH

As we previously described^24^, to determine if estrogen can cause deletions and LOH in BRCA1^mut/+^ cells, the cells were treated with 30 µM Estrogen overnight. Formaldehyde was used as a positive control. The cells were treated with 100 µM Formaldehyde for 6 h and released with fresh media overnight. All cultures were treated with Colcemid (ThermoFisher Scientific, #15212012) at final concentration of 0.1 µg/mL, following 30–60 min incubation, the cells were trypsinized according to standard procedures, incubated in 0.075 M KCl for 10 min at 37 °C and fixed in methanol-acetic acid (3:1). FISH analysis was performed on fixed cells and a 2-color probe prepared to separately interrogate the *BRCA1* gene locus. The bacterial artificial chromosome (BAC) clones used in the probe-mix were as follows: *BRCA1* (RP11-831F13; labeled with red dUTP) and 3’*BRCA1* (RP11-948G15; labeled with green dUTP). For each gene, the 3’locus served as the control. All RP11 clones were purchased from the Roswell Park Cancer Institute Genomics Shared Resource (Buffalo, NY). Probe labelling, hybridization, post-hybridization washing, and fluorescence detection were performed according to procedures established at the Molecular Cytogenetics Core Facility. Slides were scanned using a Zeiss Axioplan 2i epifluorescence microscope (Carl Zeiss Microscopy, Thornwood, NY) equipped with Isis imaging software (MetaSystems Group Inc, Waltham, MA). The entire hybridized area was scanned through 63X objective lens to assess the quality of hybridization and signal pattern. Following an initial scan, for each cell line, a minimum of 60 metaphase spreads were imaged and analyzed for deletions and hemizygous loss. Average ± Std Deviation was calculated from 2 independent experiments. Statistical analysis was conducted using Student’s *t*-test with two-tailed distribution for *p*-value calculation.

## Supplementary Information

Below is the link to the electronic supplementary material.

Supplementary Material 1

## Data Availability

The datasets used and/or analyzed during the current study are available from the corresponding author on reasonable request.
