# Supplementary material for: Endogenous and environmental factors that induce DNA replication defects and genomic instability in ER-negative heterozygous BRCA1 cells
Source: Sci Rep. 2026 Mar 27;16:10856. doi: 10.1038/s41598-026-46028-5 (PMC13039430; doi:10.1038/s41598-026-46028-5)
Supplement: Supplementary file 1 — Supplementary Material 1 [file 41598_2026_46028_MOESM1_ESM.docx]

**Supplementary Information to:**

**Endogenous and environmental factors that induce DNA replication defects and genomic instability in ER-negative heterozygous BRCA1 cells**

# Madhura Deshpande^1^*, Theodore Paniza^1^, Rebecca Brown^1^, Kate Heslin^1^, Nitya Patel^1^, Advaitha Madireddy^4^, Zev Rosenwaks^1^ and Jeannine Gerhardt^1, 2^*

^1^The Ronald O. Perelman and Claudia Cohen Center for Reproductive Medicine, Weill Cornell

Medicine, New York, NY

^2^ Department of Obstetrics and Gynecology, Weill Cornell Medicine, New York, NY

^3^ Department of Pediatric Hematology/Oncology, Rutgers University, New Brunswick, NJ

**Suppl. Fig.1**

**
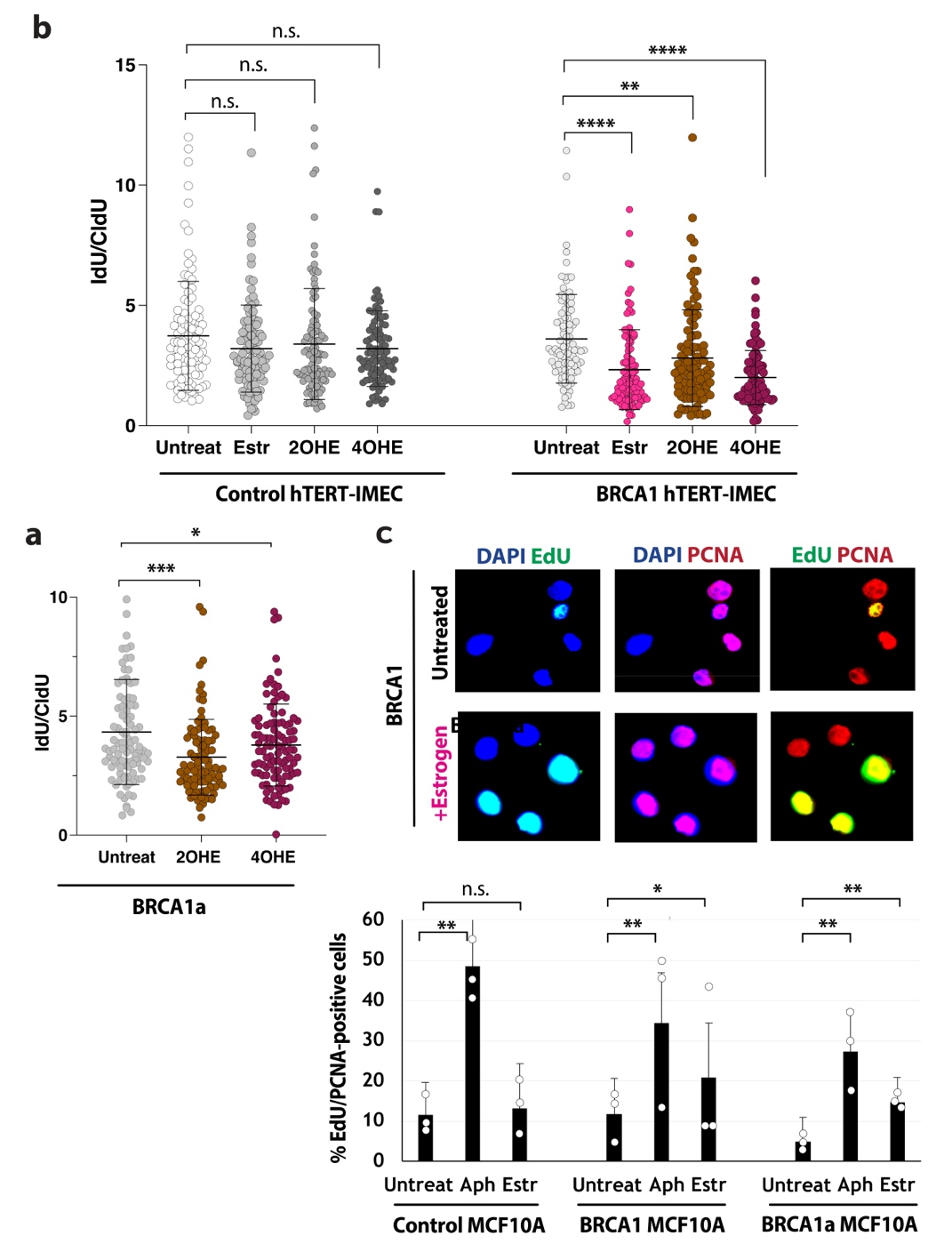
**

**Suppl. Fig.1: β-estradiol and estrogen metabolites induce replication stress in BRCA1^mut/+^ cells, related to Figure 1**. **(a-b)** DNA fiber analysis of estrogen metabolite-treated control and BRCA1^mut/+^ hTERT-IMEC cells and BRCA1a MCF10A cells. The IdU/CldU ratios were calculated. **(c)** Quantification of the percentage of cells with EdU and PCNA staining upon β-estradiol (Estr) treatment. The percentage of cells positive for both EdU and PCNA was calculated (n=100). Error bars and *p*-values (*p*= *<0.05) are indicated. Statistical analysis was conducted using two-sided Welch’s t-test for *p*-value calculation.

**Suppl. Fig.2**


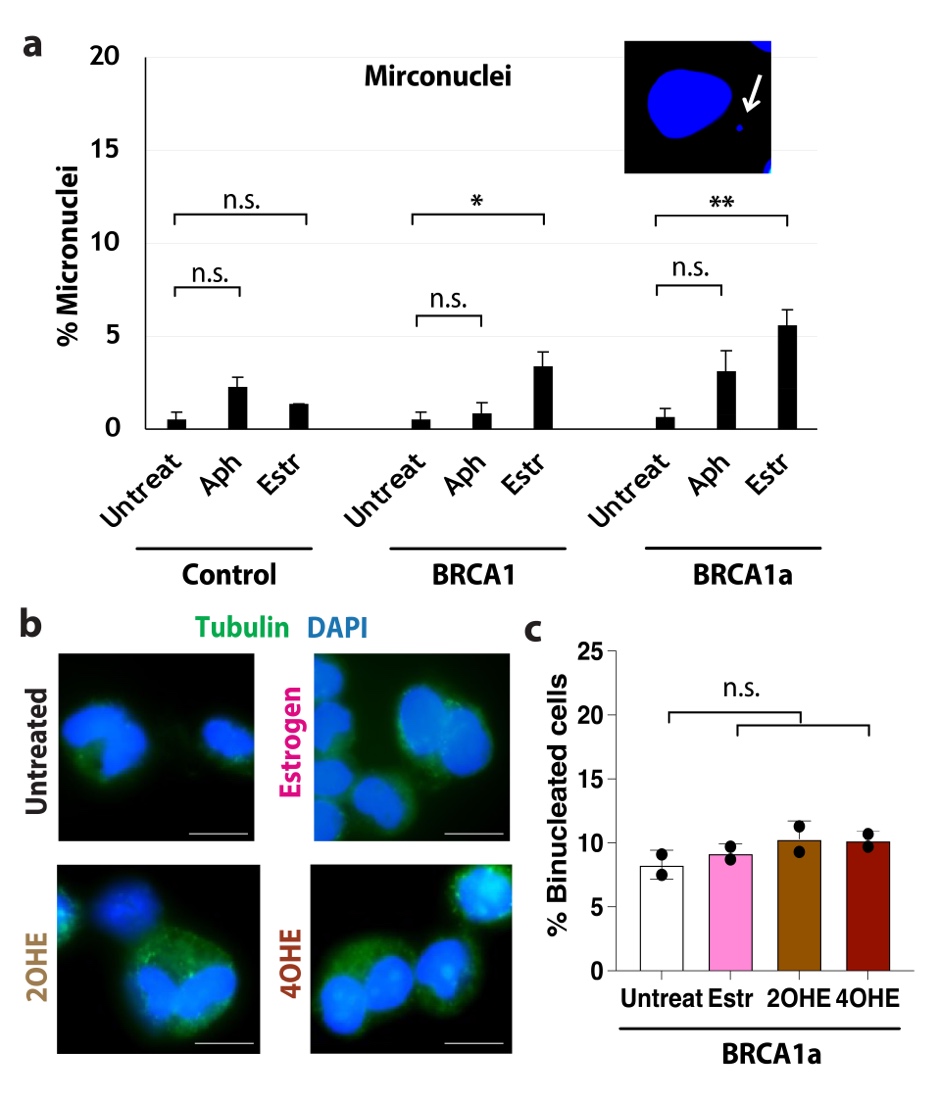


**Suppl. Fig.2: β-estradiol induces genomic instability, related to Figure 2. (a)** Analysis of the genomic instability in control and BRCA1^mut/+^ MCF10A cells treated with β-estradiol using micronuclei (MN) assay. DNA are stained with DAPI. The percentage of cells with MN (n=100) is shown, and error bars are indicated. Statistical analysis was conducted two-sided Welch’s t-test for *p*-value calculation. **b-c)** Analysis of binucleated cells in BRCA1a^mut/+^ cells. Cells were treated with 1 µM β-estradiol or estrogen metabolites for 72 h. The percentage of cells with two or more nuclei (n > 250) was calculated. Comparisons were made between treatments, and *p*-values were calculated and are indicated. Representative images are shown in (**b**). Statistical analysis was conducted using two-sided Welch’s t-test for *p*-value calculation.

**Suppl. Fig.3**

**
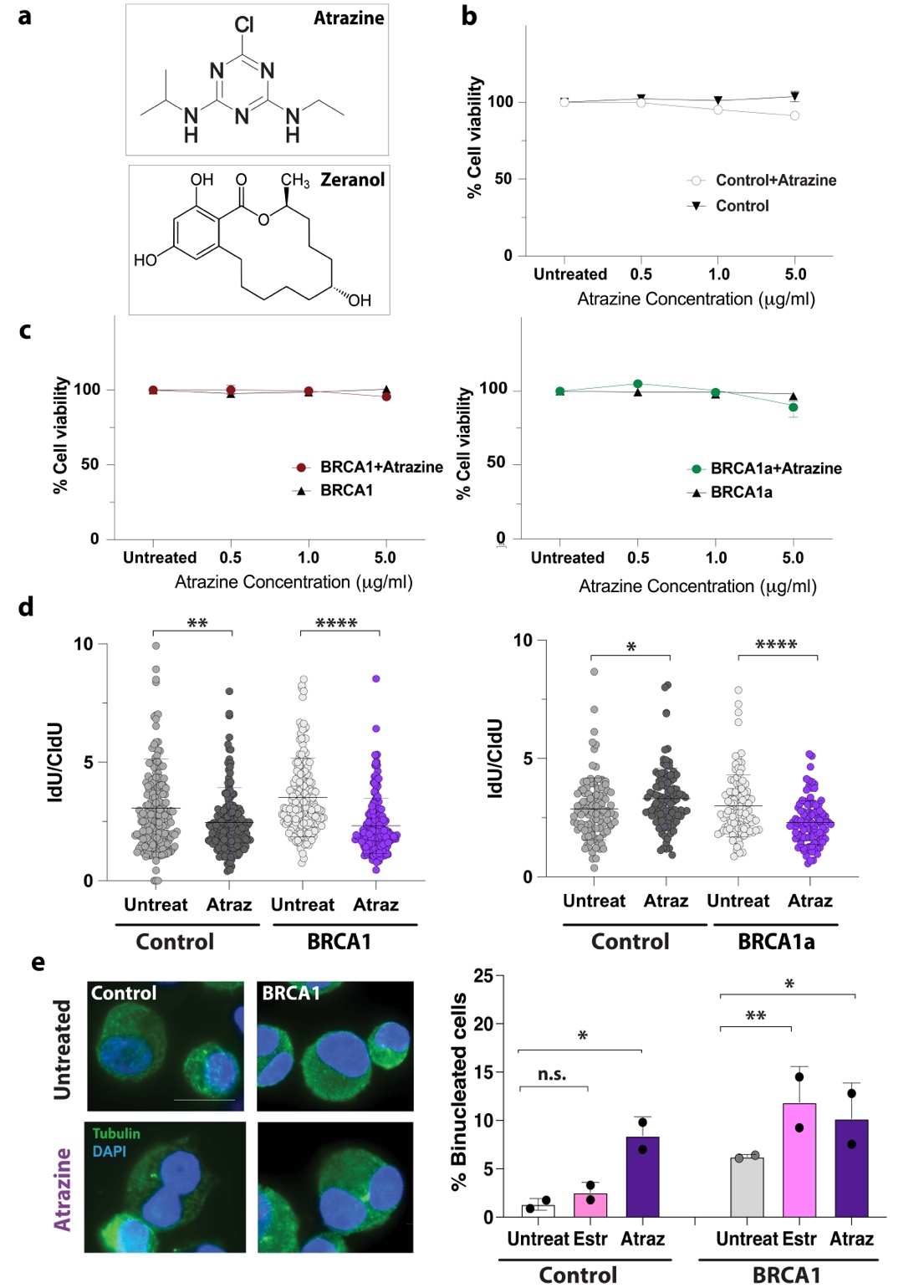
**

**Suppl. Fig.3: Atrazine causes replication stress and DNA damage in BRCA1^mut/+^ cells, related to Figure 4. (a)** Structure of Atrazine and Zeranol. **(b-c)** Analysis of the cell viability without and with Atrazine treatment in control and in BRCA1^mut/+^ cells, n=100. **(d)** DNA fiber experiments on BRCA1^mut/+^ cells treated without and with Atrazine. Analysis of the fork rate (IdU/CldU ratio) is shown. The *p*-values and error bars are indicated. **(e)** Analysis of binucleated cells in control and BRCA1^mut/+^ cells. Cells were treated with 1 μg/ml Atrazine and 1 μM β-estradiol for 72 h. The percentage of cells with two or more nuclei (n > 250) was calculated. Comparisons were made between treatments. Representative images are shown on the left. Statistical analysis was conducted using two-sided Welch’s t-test for *p*-value calculation. *p*-values are indicated: * <0.05 ** <0.005 *** <0.0005.

**Suppl. Fig.4**

**
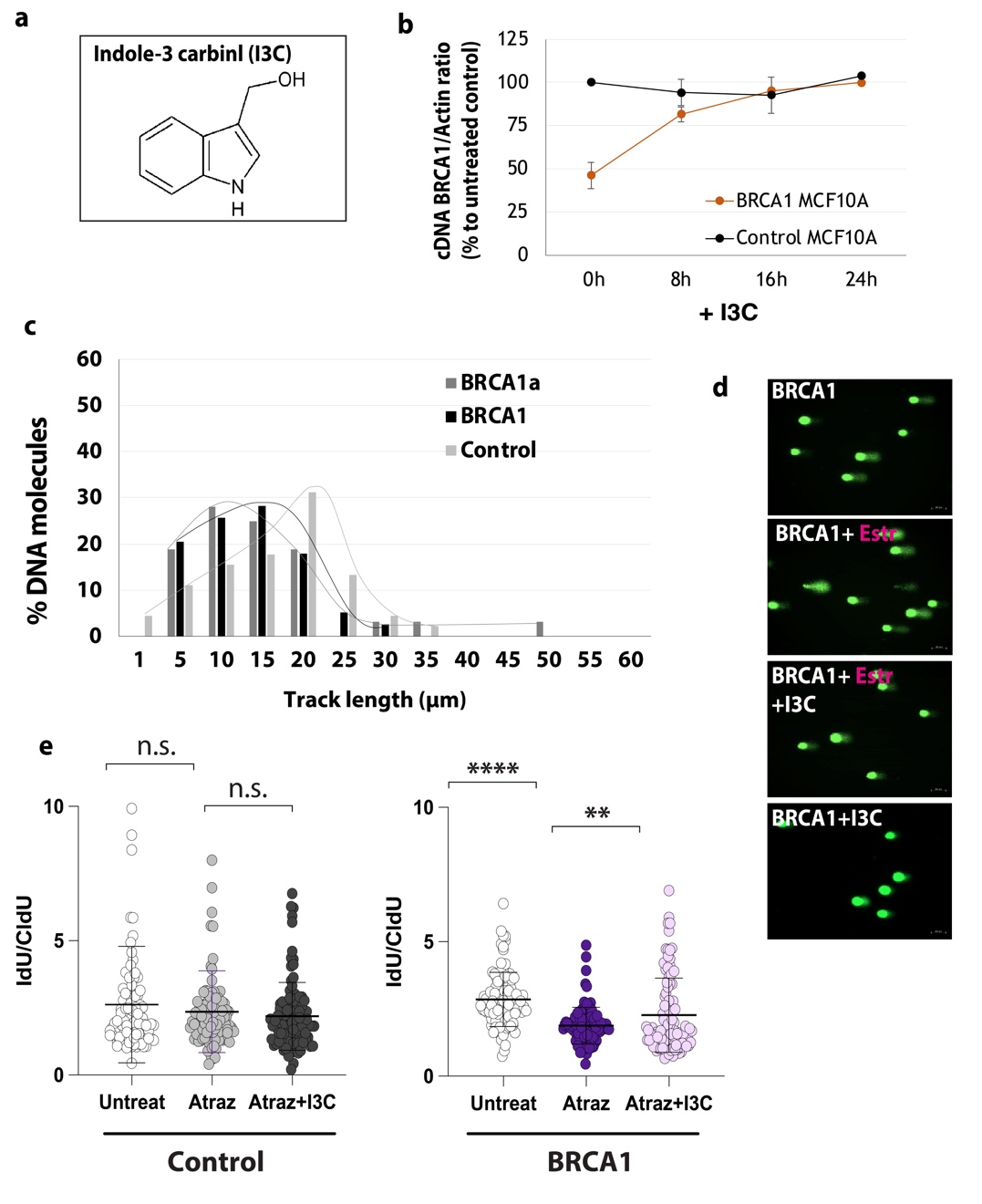
**

**Suppl. Fig.4: I3C increases BRCA1 expression and reduces replication stress in BRCA1^mut/+^ cells, related to Figure 5. (a)** Structure of Indole-3-carbinal (C_9_H_9_NO). (**b**) RT-PCR with BRCA1 or Actin primer to determine BRCA1 and Actin expression in control and BRCA1^mut/+^ cells without or with I3C treatment (8, 16 and 24h). Calculation of the ratio of BRCA1/Actin expression, which is displayed as percentage of the BRCA1 expression in untreated control cells (as described before in^45^. **(c)** DNA fiber analysis of untreated control cells. **(d)** Representative images from the analysis of the DNA breaks in BRCA1^mut/+^ cells treated either with 1μM β-estradiol (Estr) and/or 100 μM I3C using comet assay. **(e)** DNA fiber analysis of Atrazine-treated control and BRCA1^mut/+^ cells with and without 100 μM I3C. The fork rate (IdU/CldU ratio) was calculated. The *p*-values and error bars are indicated, n=100, Statistical analysis was conducted using two-sided Welch’s t-test. *p*-values are indicated: * <0.05 ** <0.005 *** <0.0005.

**Suppl. Fig.5**


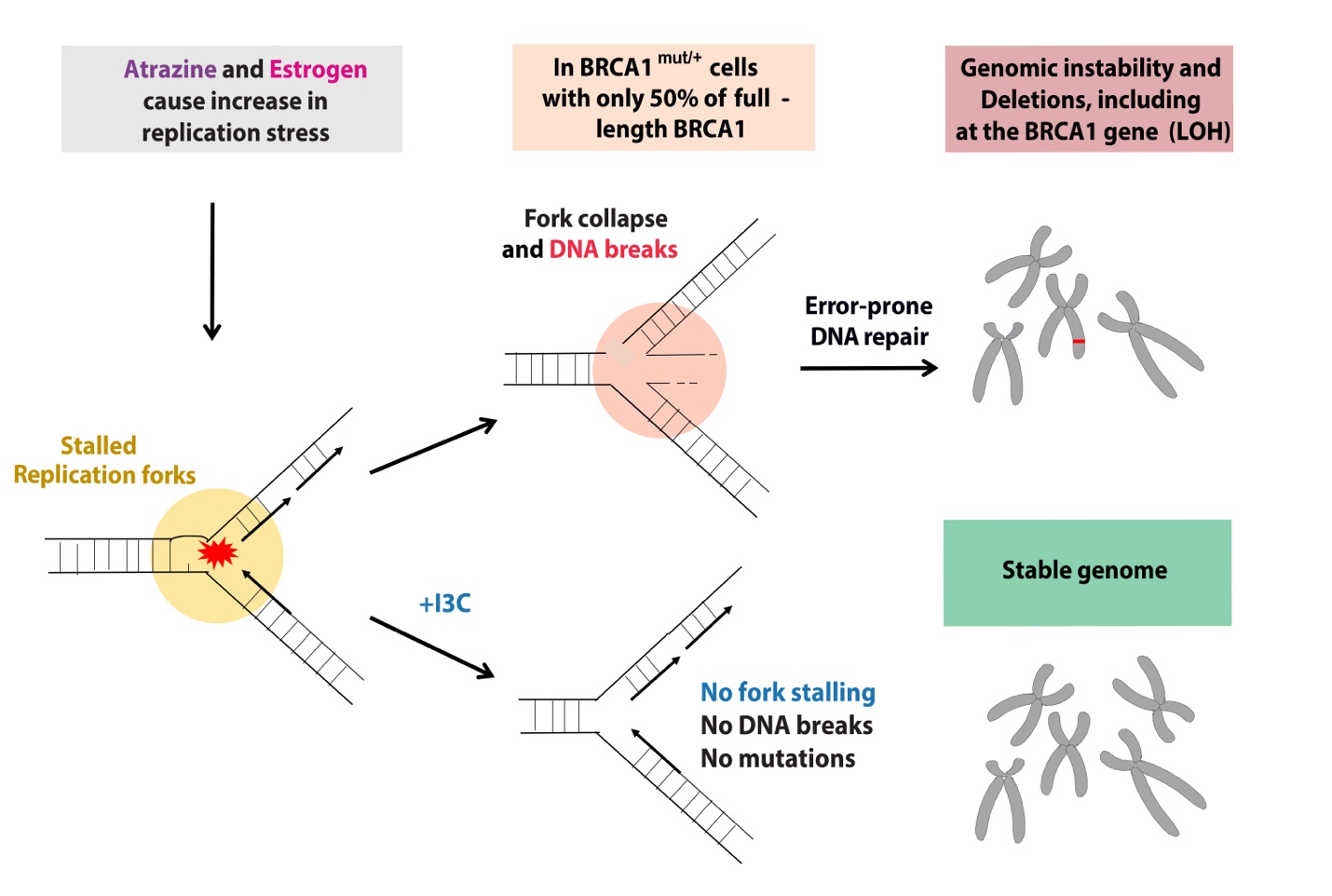


**Suppl. Fig.5: Model how β-estradiol induces genomic instability in BRCA1^mut/+^ cells.** Illustration indicating how β-estradiol and estrogen metabolites can cause genomic instability and prime cells to cause cancer initiation in BRCA1^mut/+^ carrier cells. Estrogen and estrogen metabolites form depurinating DNA adducts that leave apurinic (AP) sites, which stall the replication forks. These DNA lesions are repaired by error-prone pathway in BRCA1^mut/+^ cells, leading to mutations (including large deletions) and LOH. This instability, DNA lesions and dependence on error-prone repair pathway initiate cancer in BRCA1^mut/+^ cells. Estrogen metabolites induce cancer-like features, such as binucleation and EMT transition in BRCA1^mut/+^ cells. I3C prevents replication stress and DNA damage in BRCA1^mut/+^ cells, thus could be used as a preventive agent for BRCA1 carriers.
